# MiRNAs Which Target CD3 Subunits Could Be Potential Biomarkers for Cancers

**DOI:** 10.1371/journal.pone.0078790

**Published:** 2013-11-11

**Authors:** Fariborz Asghari Alashti, Zarrin Minuchehr

**Affiliations:** Department of Bioinformatics, National Institute of Genetic Engineering and Biotechnology, Tehran, Iran; Niels Bohr Institute, Denmark

## Abstract

**Background:**

T-cells play an important role in the immune response and are activated in response to the presentation of antigens bound to major histocompatibility complex (MHC) molecules participating with the T-cell receptor (TCR). T-cell receptor complexes also contain four CD3 (cluster of differentiation 3) subunits. The TCR-CD3 complex is vital for T-cell development and plays an important role in intervening cell recognition events. Since microRNAs (miRNAs) are highly stable in blood serum, some of which may target CD3 molecules, they could serve as good biomarkers for early cancer detection. The aim of this study was to see whether there is a relationship between cancers and the amount of miRNAs -targeted CD3 molecules.

**Methods:**

Bioinformatics tools were used in order to predict the miRNA targets for these genes. Subsequently, these highly conserved miRNAs were evaluated to see if they are implicated in various kinds of cancers. Consequently, human disease databases were used. According to the latest research, this study attempted to investigate the possible down- or upregulation of miRNAs cancer patients.

**Results:**

We identified miRNAs which target genes producing CD3 subunit molecules. The most conserved miRNAs were identified for the CD3G gene, while CD247 and CD3EAP genes had the least number and there were no conserved miRNA associated with the CD3D gene. Some of these miRNAs were found to be responsible for different cancers, following a certain pattern.

**Conclusions:**

It is highly likely that miRNAs affect the CD3 molecules, impairing the immune system, recognizing and destroying cancer tumor; hence, they can be used as suitable biomarkers in distinguishing cancer in the very early stages of its development.

## Introduction

More than 30% of the human genome is controlled by miRNAs, regulating important biological processes [1]. These short non-coding RNAs (21–23 nucleotides long) are considered to be associated with most cancers [2], and have thus attracted the attention of many researchers. miRNAs are bound to the three prime untranslated regions (3′UTR) of target messenger RNAs (mRNAs) before they can be translated to special proteins by making genes off and on [3], [4]. They have key roles in lots of cellular processes such as cell proliferation [4], cell division [5], apoptosis [6], differentiation, self-renewal [7], stress response and cancer [5]. Down-regulation of some miRNAs (for example miR-34) which is the result of reduced expression, leads to an increase in cells in certain types of cancers [8]. The expression of the majority of miRNAs is reduced in human cancers ([9], [10], [11]), which is in contrast to the overxpression certain miRNAs leading to the progression of tumorigenesis [11]. Therefore, miRNAs can act as both tumor suppressors or oncogenes, and their expression may provide a predictive diagnostic value [11]. miRNAs also play an important role in the development of the immune system in which their proper expression can lead to advanced growth, that may otherwise, lead to an interruption of immunity [12].

There is strong evidence that has verified the effectiveness of miRNAs in the immune system. Accordingly, miRNAs can affect the development of B cells [13] and T cells. They can also increase monocyte and neutrophil levels, and are responsile for antibody switching and the release of inflammatory mediators, which are released by the immune cells during times when harmful agents invade the body [14]. miRNAs are stable and consistent in individuals of the same species and can be used as potential biomarkers for the recognition of various cancers and other diseases [15], [16]. miRNA signatures in the blood are similar in men and women in different ages [17]. Some findings suggest that they can be accepted as suitable biomarkers, for instance, miR-29a and miR-92 plasma levels were found to be different in controls and colorectal cancers [18]. In fact, an increase in the levels of miR-21, miR-92 and miR-93 could be regarded as a clinical biomarker of ovarian cancer [19].

Since, the thymus is the principal organ responsible for T cell maturation [20], which continues to grow between birth and puberty, and that thymic activity (T-cell output) is the most active before puberty and undergoes atrophy in aging, inevitably weakening the immune response [21]; it can thus be inferred that there is a positive correlation between cancer and age [22]. Therefore, it would not be very surprising to think of the immune system as key to cancer therapy.

The immune system can recognize and omit tumors which express antigens that are not observed in normal cells. So such antigens are considered as foreign by the immune system, which ultimately attacks and destroys them [23] using killer T cells, and occasionally with the support of the helper T cells [24]. These antigens function as viral forms by acting on the MHC class I molecules; killer T cells recognize tumors as nonstandard cells [25]. Natural killer cells are another type of leukocyte that attack tumor cells and cells infected by viruses. They kill cells by using lower levels of MHC class I molecules persent on their surface when compared to normal cells [26]. The T cell receptor or TCR, which is a molecule found on the surface of the T-cells, can help recognize antigens bound to MHC [27].

The TCR heterodimer is responsible for ligand recognition. When it assembles with four invariant proteins (CD3-gamma, -delta, -epsilon and -zeta), it holds a characteristic sequence motif associated with the immunoreceptor tyrosine-based activation motifs (ITAMs), which are important for signal transduction in immune cells. The TCR_CD3 complex interaction plays an important role in intervening cell recognition events [28].

The key role of miRNAs in the immune system has been implicated by scientists; for example, miR-150 affects some mRNAs which are important for development of pre- and pro-B cells. So every change in these miRNAs may obstruct the development of the immune system [29] [14].

miRNAs target mRNAs at specific sites to cause translational repression and inhibit translation or induce cleavage of the massage [30]. There are some microRNA target gene databases such as StarBase [31], miRwalk [32], targetScan [32] etc. which help in finding the best miRNA target gene.

In accordance with the stability of miRNA in serum and the importance of the role of CD3 molecules in recognition of antigens, this study attempted to focus on the miRNAs that target CD3 proteins which could be ultimately used as reasonable biomarkers in the detection of cancers.

## Methods

There are four CD3 subunits including CD3-Gamma, CD3-Delta, CD3-Epsilon and CD3-Zeta that bind to the TCR, leading to the formation of the TCR.CD3 complex, which plays an important role in T cell receptor function and T cell development [33]. In order to recognize the genes producing these subunits, the Ensembl genome database release 70 updated - January 2013, (http://asia.ensembl.org/Homo_sapiens/Info/Index/) was used [34]. In this database the name and ID of genes, which coded for molecules in humans, were derived. Using the TargestScan Human database release 6.2 (http://www.targetscan.org/(updated -june 2012)) [35], and choosing broadly conserved, conserved and poorly conserved microRNA family options, we obtained miRNAs that were able to target these genes. It is worth mentioning that this study only considerd the results of conserved microRNA families, but no other genes.

Subsequently, every miRNA of the conserved microRNA family has been investigated separately. In order to find out in which type of cancer, these miRNAs play an important role, we used the Human miRNA & Disease Database(HMDD) (http://202.38.126.151/hmdd/mirna/md/(September 2012)) [36]. In this database, diseases related to miRNAs and their role were identified. A search for miRNAs was also performed by browsing the miRCancer Database (http://mircancer.ecu.edu/browse.jsp (January 2012)) [37]. In this database, miRNAs were searched according to their name, eventually leading to identification of cancers they were responsible for. This database also showed whether miRNAs are upregulated or downregulated.

## Results

The TCR-CD3 complex is a multimeric structure present on the T-cells surface. Human CD3 consists of four subunits: CD3-Gamma, CD3-Delta, CD3-Epsilon and CD3-zeta. To know the name and features of genes which are involved in expressing CD3 subunit molecules, the Ensembl database was used, and one gene for each subunit was found and identified as; CD3G, CD3D, CD3EAP and CD247, respectively (Table 1).

**Table 1:** The name and other characteristics of genes which are expressed to produce CD3 subunit molecules.

Protein Name	Gene symbol	Gene ID	Location
CD3G molecule, gamma	CD3G	ENSG00000160654	11∶118215059–118225876∶1
CD3D molecule, delta	CD3D	ENSG00000167286	11∶118209669–118213459:−1
CD3E molecule, epsilon associated protein	CD3EAP	ENSG00000117877	19∶45909467–45914024∶1
CD3 molecule, zeta	CD247	ENSG00000198821	1∶167399877–167487847:−1

For each of the four genes involved in producing CD3 subunits, the TargestScanHuman database (release 6.2-June 2012) was used [35] to identify and predict miRNAs which could target these genes. In this study, only highly conserved miRNA families have been considered and poorly conserved miRNA families have been omitted. For the CD3G gene, 21 miRNAs were predicted that target this gene (Table 2). One miRNA was predicted for the CD3EAP gene (responsible for producing the CD3E molecule, epsilon) (Table 3), three miRNAs for the CD247 gene (responsible for producing the CD3 molecule, zeta) in both transcripts of NM_000734 (1041 nt) and NM_198053 (1041 nt) (Table 4). miRNA were not predicted for the CD3D gene (responsible for producing the CD3 molecule, delta) in both transcripts of NM_000732 (88 nt) and NM_001040651 (88 nt).

**Table 2:** miRNAs which were predicted to target CD3G (broadly conserved).

Predicted miRNAs	Target region of gene pairing with miRNA
Has-miR-1200	Position 2–8 of CD3G 3′ UTR
Has-miR-378d	Position 17–23 of CD3G 3′ UTR
Has-miR-378	Position 17–23 of CD3G 3′ UTR
Has-miR-378e	Position 17–23 of CD3G 3′ UTR
Has-miR-378i	Position 17–23 of CD3G 3′ UTR
Has-miR-378c	Position 17–23 of CD3G 3′ UTR
Has-miR-422a	Position 17–23 of CD3G 3′ UTR
Has-miR-378h	Position 17–23 of CD3G 3′ UTR
Has-miR-378b	Position 17–23 of CD3G 3′ UTR
Has-miR-378f	Position 17–23 of CD3G 3′ UTR
Has-miR-3690	Position 18–24 of CD3G 3′ UTR
Has-miR-619	Position 19–25 of CD3G 3′ UTR
Has-miR-4446-3p	Position 94–101 of CD3G 3′ UTR
Has-miR-2909	Position 96–103 of CD3G 3′ UTR
Has-miR-4777-5p	Position 103–109 of CD3G 3′ UTR
Has-miR-136	Position 132–139 of CD3G 3′ UTR
Has-miR-515-5p	Position 134–140 of CD3G 3′ UTR
Has-miR-4659a-3p	Position 137–144 of CD3G 3′ UTR
Has-miR-4659b-3p	Position 137–144 of CD3G 3′ UTR
Has-miR-494	Position 203–209 of CD3G 3′ UTR
Has-miR-593	Position 405–412 of CD3G 3′ UTR

**Table 3:** miRNAs which were predicted to target CD3EAP (broadly conserved).

Predicted miRNAs	Target region of gene pairing with miRNA
Has-miR-138	Position 95–101 of CD3EAP 3′ UTR

**Table 4:** miRNAs which were predicted to target CD247 (broadly conserved).

Predicted miRNAs	Target region of gene pairing with miRNA
Has-miR-761	Position 410–416 of CD247 3′ UTR
Has-miR-214	Position 410–416 of CD247 3′ UTR
Has-miR-3619-5p	Position 410–416 of CD247 3′ UTR

Each of the predicted miRNAs were then reviewed in related research papers to see if these miRNAs were down-regulated or up-regulated in tumor tissues and the blood serum of cancer patients.

The results showed that has-miR-378, which was a miRNA predicted for CD3G, had increased in all blood sera belonging to patients suffering from castration resistant prostate cancer (CRPC) [38], renal cell carcinoma (RCC) [16], [39] and gastric cancer (GC) [40]; besides, cancer tissues in patients with bladder cancer (BC) [41], carcinoma basal cells [42], colorectal cancer (CRC) [43], oral carcinoma [44], laryngeal carcinoma [45] and gastric cancer [46]. This was in contrast to reduced miRNA level in adjacent non-tumoral tissues. Has-miR-422a, has-miR-593 and has-miR-494 which represented other miRNAs predicted for CD3G also showed a similar decrease in levels in tumor tissues. For instance, reduction in levels were observed for miR-422a in patients with laryngeal carcinoma [45] and colorectal cancer [43], miR-593 in esophageal cancer patients [47] and miR-494 in cholangiocarcinoma (CCA) [48], lung cancer [49] and gastrointestinal tumor [50]. The above observations were in agreement with the data of this study. Except for miR-515 and miR-136, which were only cited once (oral squamous cell [44] and lung cancer [51] respectively), which showed an increasing number of miRNAs in tumor tissues), no other literature is available regarding the number of other miRNAs predicted for targeting CD3G in tumor tissues or blood sera of cancer patients (Table 5).

**Table 5:** The status of miRNAs from the first group which affect the CD3G gene responsible for the CD3-gamma subunit in cancer tissues relative to adjacent normal tissues.

miR-ID	Profile	Cancer	reference
Has-miR-378	down-regulated	bladder cancer	[41]
	down-regulated	carcinoma basal cells	[42]
	down-regulated	CRC tumor	[43]
	down-regulated	oral carcinoma	[44]
	down-regulated	laryngeal cancer	[45]
	down-regulated	stomach neoplasm	[46]
Has-miR-422a	down-regulated	laryngeal neoplasm	[45]
	down-regulated	colorectal neoplasm	[43]
Has-miR-593	down-regulated	esophageal cancer (EC)	[47]
Has-miR-494	down-regulated	cholangio carcinoma (CCA),	[48]
	down-regulated	lung cancer	[49]
	down-regulated	gastrointestinal neoplasm	[50]
Has-miR-515	up-regulated	oral squamous cell carcinoma	[44]
Has-miR-136	up-regulated	lung neoplasm	[51]

The only miRNA predicted for targeting the CD3EAP gene, involved in CD3E expression, was hsa-miR-138. Studies showed that levels of this miRNA had decreased in tumour tissues of patients suffering from colorectal cancer [52], hepatocellular carcinoma (HCC) [53], tongue squamous cell carcinoma (TSCC) [54], papillary thyroid carcinoma [55], head and neck squamous cell carcinoma (HNSCC) [56] and squamous cell carcinoma (SCC) [57] (Table 6).

**Table 6:** The status of miRNA from the second group which affect the CD3EAP gene responsible for the CD3-epsilon subunit in cancer tissues relative to adjacent normal tissues.

miR-ID	Profile	Cancer	reference
Has-miR-138	down-regulated	colorectal cancer	[52]
	down-regulated	HCC	[53]
	down-regulated	TSCC	[54]
	down-regulated	papillary thyroid carcinoma	[55]
	down-regulated	HNSCC	[56]
	down-regulated	SCC	[57]

Studies on the predicted miRNAs targeting CD247, which were involved in the expression of the CD3-zeta molecule, showed that there were three miRNAs which targeted the gene. Except for hsa-miR-214, no reports have been cited so far regarding the levels of miR-761 and miR-3619-5p in tumor tissues. Most of the data obtained in this study showed that miR-214 levels decreased in tumor tissues when compared to its relative normal tissue (Table 7). miR-214 levels had decreased in tumor tissues of patients suffering from esophageal squamous cell carcinoma (ESCC) [58], breast cancer [59], cervical cancer [60], hepatocellular carcinoma (HCC) [61] [62], squamous cell carcinoma (SCC) [63], cholangiocarcinoma metastasis [64] and primary central nervous ystem lymphoma (PCNSL) [65]. In contrast, gastric cancer [66], ovarian cancer [67] and pancreatic cancer [68] are examples of cancers in which miR-214 levels increased (Table 7). With regard to the miR-214 copy number in blood serum studies, an increase in miRNA levels was observed in the blood sera of patients with prostate [69] and breast [70] cancers (Table 8).

**Table 7:** The status of miRNAs from the third group which affect the CD247 gene responsible for the CD3-zeta subunit in cancer tissues relative to adjacent normal tissues.

miR-ID	profile	Cancer	reference
has-miR-214	Down-regulated	ESCC	[58]
	Down-regulated	breast cancer	[59]
	Down-regulated	cervical cancer	[60]
	Down-regulated	SCC	[63]
	Down-regulated	HCC	[61]
	Down-regulated	HCC	[62]
	Down-regulated	cancer metastasis	[64]
	Down-regulated	PCNSL	[65]
	Up-regulated	pancreatic cancer	[68]
	Up-regulated	Gastric cancer	[66]
	Up-regulated	ovarian cancer	[67]

**Table 8:** The status of miRNAs in blood serum of cancer patients relative to normal patients.

miR-ID	profile	cancer	reference
has-miR-214	up-regulated	breast neoplasm	[70]
	up-regulated	prostate cancer	[69]
has-miR-378	up-regulated	CRPC	[38]
	up-regulated	RCC	[39]
	up-regulated	RCC	[16]
	up-regulated	gastric cancer	[40]

## Discussion

miRNAs can act as tumor oncogenes and supressors and negatively regulate gene expression [71]. This task can be performed through degradation of mRNA or repression of translation [72]. Many recent studies have described miRNAs as key regulators of the processes in the immune system, which have critical roles in the induction, function and maintenance of the regulatory T-cell lineage [73]. The TCR–CD3 complex, which is a complex transmembrane receptor, plays a decisive role in the immune system [74]. Four invariant chains, physically associated with TCR, include: CD3-gamma, -delta, -epsilon and -zeta [75]. miRNAs have previously been identified as powerful biomarkers in a variety of cancers [16], [17], [18], [76], [77], [78], and in this study, the bioinformatics analysis predicted miRNAs which targeted genes expressing the CD3 subunits, including CD3-gamma, Cd3-delta, CD3-epsilon and CD3-zeta. Our aim was to identify miRNAs which affect subunits of the immune system and to validate them by reviewing the studies which had previously been carried out, so as to evaluate them as potential biomarkers for diagnosis or early detection of cancers. In In this study, the TargetScanHuman database identified13 miRNAs (miR-1200, miR-378d,e,i,c,h,b,f, miR-422a, miR-3690, miR-619, miR-4446-3p, miR-2909, miR-4777-5p, miR-136, miR-515-5p, miR-4659a,b-3p, miR-494 and miR-593) which were conserved and similar to the CD3G gene. One miRNA (miR-138) was found to be conserved and similar to the CD3EAP gene and three conserved miRNAs (miR-761, miR-214 and miR-3619-5p) were shown to be similar to the CD247 gene. However, no miRNAs were found to be conserved and similar to the CD3D gene [35]. From the first group only 6 miRNAs (miR-378, miR-422a, miR-593 and miR-494, miR-515 and miR-136), from the second group one miRNA (miR-138) and from the third group only one miRNA (miR-214) were identified and studied, based on their levels in tissues or blood sera of different cancer patients. miR-378, miR-422a, miR-593 and miR-494 from the first group, which affected the CD3G gene (responsible for the CD3-gamma subunit), and were down-regulated in cancer tissues, when compared to adjacent normal tissues (Table 5), whereas miR-378 was up-regulated in blood sera of cancer patients (Table 8). With respect to a number of studies, miR-515 and miR-136 were up-regulated in cancer tissues (Table 5). miR-138 (Table 6) from the second group (predicted for targeting the CD3EAP gene responsible for the CD3-epsilon subunit) was down-regulated, and miR-214 from the third group (predicted for targeting the CD247 gene responsible for the CD3-zeta subunit), was down-regulated in cancer tissues investigated in most of the studies. However, theses were reported to be up-regulated in only a few research papers (Table 7). miRNAs are currently being considered as down-regulated tumor suppressors and up-regulated oncomiRs [52], [79]. Based on the case that miR-378 and miR-214 were up-regulated in the blood serum of cancer patients (Table 8) and down-regulated in the tumor tissues; therefore, it can be concluded that they can function as upregulated oncomiRs miRNAs on subunits of the TCR-CD3 complex.

## Conclusion

Since miR-378, miR-422a, miR-593, miR-494, miR-138 and miR-214 could target the CD3 subunits, some of which have been studied in different cancers and have been considered as biomarkers to detect cancer at the early stages, it is then highly likely that miRNAs damage the immune system so that it cannot distinguish cancer cells. Hence, their presence in the blood serum as biomarkers for early diagnosis of cancer could help us to understand the disability of immune system. Although this study looks bioinformatically reliable, more laboratory studies are needed to support these findings.
